# Electroactive Bacteria in Natural Ecosystems and Their Applications in Microbial Fuel Cells for Bioremediation: A Review

**DOI:** 10.3390/microorganisms11051255

**Published:** 2023-05-10

**Authors:** Gian Luigi Garbini, Anna Barra Caracciolo, Paola Grenni

**Affiliations:** 1 Department of Ecology and Biological Sciences, Tuscia University, 01100 Viterbo, Italy; gianluigi.garbini@irsa.cnr.it; 2 Water Research Institute, National Research Council, Montelibretti, 00010 Rome, Italy; grenni@irsa.cnr.it; 3 National Biodiversity Future Center (NBFC), 90133 Palermo, Italy

**Keywords:** electrogenic bacteria, exoelectrogenic bacteria, extracellular electron transfer mechanisms, bio-recovery, water, wastewater, soil, sediment

## Abstract

Electroactive bacteria (EAB) are natural microorganisms (mainly *Bacteria* and *Archaea*) living in various habitats (e.g., water, soil, sediment), including extreme ones, which can interact electrically each other and/or with their extracellular environments. There has been an increased interest in recent years in EAB because they can generate an electrical current in microbial fuel cells (MFCs). MFCs rely on microorganisms able to oxidize organic matter and transfer electrons to an anode. The latter electrons flow, through an external circuit, to a cathode where they react with protons and oxygen. Any source of biodegradable organic matter can be used by EAB for power generation. The plasticity of electroactive bacteria in exploiting different carbon sources makes MFCs a green technology for renewable bioelectricity generation from wastewater rich in organic carbon. This paper reports the most recent applications of this promising technology for water, wastewater, soil, and sediment recovery. The performance of MFCs in terms of electrical measurements (e.g., electric power), the extracellular electron transfer mechanisms by EAB, and MFC studies aimed at heavy metal and organic contaminant bioremediationF are all described and discussed.

## 1. Introduction

Electrogenic or electroactive bacteria (EAB) are natural microorganisms able to generate electricity through various metabolic processes. Although they have only recently been exploited for practical applications, the first author who described electricity generation by a microorganism was the Botanics Professor M.C. Potter at Durham University in 1911 [1]. He observed that immerging a platinum electrode into a liquid medium with a yeast and a bacterial suspension, in the presence of glucose, produced a voltage of 0.3–0.5 V. Subsequently, further developments in this topic were performed by Barnet Cohen in 1931, who was able to produce 35 V connecting a number of microbial half fuel cells in series mode [2]. These pioneer studies were the basis for the development of electromicrobiology, a new discipline, which deals with the electron exchange between microorganisms and external electronic devices, involving microbial functions that can be involved in the emerging field of bioelectronics [3].

Bennetto and Allen [4] from King’s College (London) designed the “microbial fuel cells” currently used: a two chamber bioreactor with a carbon felt electrode separated by an ion exchange membrane. Kim et al. [5] identified the first electrogenic bacterium, *Shewanella oneidensis* (ex *Shewanella putrefaciens*), which is a Fe(III)-reducing organism and facultative aerobic/anaerobic bacterium [6].

Subsequently, electrochemical activity was observed in several different bacterial strains, and various microbial fuel cells were designed and tested using pure cultures and enriched mixed cultures. Bond and Lovley discovered *Geobacter sulforeducens*, which can effectively increase the performance of a microbial fuel [7]. The physiology, ecology, and applications of this electrogenic bacterium were described by Lovley et al. [8]. *Geobacter sulfurreducens* PCA and KN400 strains and *Shewanella oneidensis* strain MR-1 are considered the model organisms in microbial electrochemistry [9].

In the last few years, interest in electrogenic bacteria has been growing in view of their potential applications in green technologies dealing with renewable energy and environmental management [10]. The study of the functioning and identification of these microorganisms is only possible using an interdisciplinary approach (combining e.g., microbial ecology, energy engineering, biotechnology and bioinformatics) and application of the newest sequencing methods and metagenomic approaches. Thanks to the latter, several microbial groups (including non-cultivable ones) involved in bioelectrochemical processes are being identified. Because of their peculiar characteristics, electrogenic bacteria have been exploited in the last decade for biotechnological applications, such as bioelectrochemical systems (BESs). BESs are electrical devices that rely on electrogenic bacterial activity [11] and include various tools with different purposes [12]. BESs are able to reduce pollutants, recycling elements, synthesizing new products, and generating electricity [13]. They can also be used for biodegradation/bioremoval of several contaminants [14,15,16]. BES are in line with the circular economy model, because waste can be used as the fuel material, converting it to energy [17].

There are various BES types, which include different technologies, such as microbial fuel cells (MFC), microbial electrolysis cells (MEC), and microbial electrosynthesis (MES) [11]. This review focuses on prokaryotic groups directly or indirectly involved in the production of electricity within MFC, and examples of application for bioremediation are reported.

MFCs are BES that transform organic waste into electricity through microbial electrochemical reactions catalysed in the anodic and cathodic regions [18,19,20]. Electrogenic bacteria develop a biofilm, under anaerobic conditions, on the anode (mainly located at the bottom of an MFC) and catabolize (oxidize) organic compounds (including several contaminants), producing and releasing protons (H^+^), electrons (e^−^), and carbon dioxide (CO_2_). The electrons released by bacteria are transferred to the anode, and then through an external circuit to the cathode, where oxygen acts as the electron acceptor. Protons flow from the anode to the cathode through the electrolyte (water or soil/sediment), which also provides organic matter used by the bacterial communities [21].

## 2. Electromicrobiomes in Natural Ecosystems

Microbial communities (mainly prokaryotic cells, but in some cases also fungi) that live in natural environments where they can form biofilms, interacting electrically with each other and/or their extracellular environment, are named “electromicrobiomes” [22].

These microorganisms are widely distributed in natural ecosystems [22,23] and have been found in soil, water, sediment, intestinal systems, surfaces of corroding metals, and digesters. Moreover, electromicrobiomes have been detected in extreme environments [9,24]. For example, Yamamoto et al. [25] isolated a novel bacterium, belonging to the genus *Thiomicrorhabdus*, from a thermal vent; it has multiheme cytochrome-c proteins involved in extracellular electron transfer. Interestingly, Ren et al. [26] also found several electroactive bacteria genera in varnish rock, a dark-coloured coat rich in Fe/Mn forming on rock surfaces, which it considered an extreme environment [24].

Recent works reported electroactive bacteria in freshwater ecosystems, such as rivers or lake sediments [27,28,29]. Moreover, other authors found EAB in salt marsh [22], in mudflat marine sediment, and brackish ecosystems [30,31]. For example, in a mangrove sediment, typically rich in organic matter and anoxic conditions, these bacteria completely oxidized organic matter to CO_2_ using electron acceptors such as Mn(IV) and Fe(III) [32,33]. Although EAB have been investigated in marine and fresh water sediments, soil ecosystems have not been well explored so far, despite having a biodiversity higher than water/sediment. Most studies have focused on microbial communities in paddy soils [34,35]. Electrogenic bacteria were also found in plant tissue as endophytes in sweet potato roots *(Dioscorea esculenta*) and angelica stems *(Angelica sinensis*) [36]. The human gut has also been demonstrated to be a suitable environment for exoelectrogenic bacteria. In fact, Tahernia et al. [37] identified five gut bacteria with extracellular electron-transfer capabilities. Table 1 reports a list of electroactive bacteria found in various natural or anthropic habitats.

## 3. Extracellular Electron Transfer Mechanisms

Electroactive bacteria capable of extracellular electron transfer can also be termed exoelectrogenic bacteria, exoelectrogens, electrochemically active bacteria, anode respiring bacteria, or electricigens [57]. Currently, more than 100 microorganisms have been described as electroactive bacteria able to perform extracellular electron transfer (EET) [9]. They interact electrically with other microbial species, minerals, or soluble extracellular electron acceptors and donors that cannot enter inside a cell [57,58]. Prokaryotic cells coordinate their development, activity and mobility in these systems with advantageous cell-to-cell interactions. As a result, biofilms that are complex and have a highly organized multicellular and multispecies structure are formed [59]. In biofilms, cells are linked together and embedded in a matrix primarily composed of proteins, nucleic acids, and carbohydrate polymers [60]. Cell-to-cell contact and transfer of electrons is facilitated by extracellular polymeric compounds (EPS) [61].

Paquete et al. [61] summarized the main processes involved in electroactive microorganism interactions such as quorum sensing, coordinated by the expression of genes activated by a specific cell density and various environmental factors; outer-membrane vesicles, which make possible cell communication between cells and EET; physical interactions between neighbouring cells via nanotubes, type IV pili, multiheme c-type cytochromes, cytochrome-nanowire systems; and small diffusible metabolites (e.g., hydrogen, formate or flavins), which enable electron transfer between cells without a cell-to-cell contact [61].

Overall, electroactive microorganisms use a network of redox and structural proteins to transfer electrons between their plasma membranes and extracellular minerals. In a few model microorganisms, some of these mechanisms and proteins have been described. In particular, Shi et al. [58] reported that these proteins generally cooperate in building pathways that physically and electrically link redox reactions of metal ions associated with external minerals with intracellular metabolic processes. For example, the EET pathways of the gram-negative Proteobacteria *Geobacter sulfurreducens* and *Shewanella oneidensis* have been extensively investigated [62]. These bacteria are included among dissimilatory metal-reducing microorganisms, a group of microorganisms (comprising both *Bacteria* and *Archaea*) that can perform anaerobic respiration utilizing a metal as a terminal electron acceptor [63]. The most common metals used for these reactions are Fe(III) and Mn(IV), which are reduced to Fe(II) and Mn(II), respectively [58,64,65,66]. Microbial strategies for EET have evolved over billions of years [10], and electrons can be transferred from microorganisms to multivalent metal ions, which are associated with minerals and vice versa [58].

**Figure 1:**
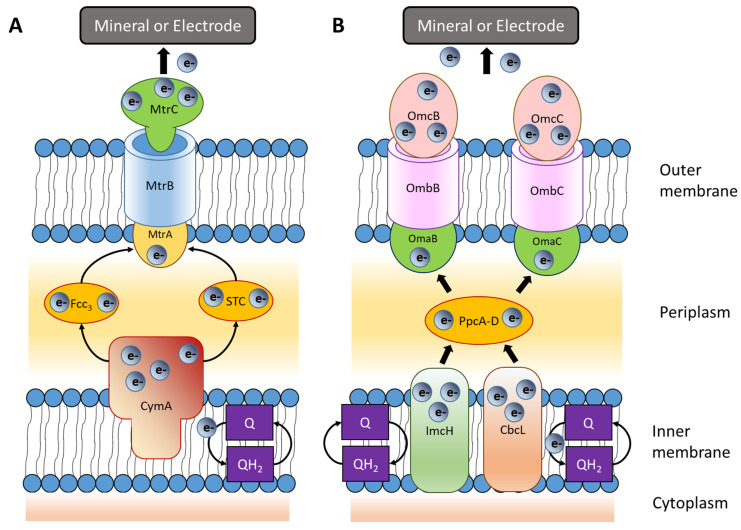
EET pathways of (**A**) *Shewanella oneidensis:* the quinol oxidases in the inner plasmic membrane transfer electrons from reduced quinols (QH_2_) to the tetraheme c-type cytochrome (CymA) bound into the membrane. In turn, CymA transfers electrons to periplasmic cytochromes (fumarate reductase, Fcc_3_ (also known as FccA), and tetraheme cytochrome, STC). In the outer membrane, the c-type cytochromes (MtrCAB) form conduits through membrane porins and transfers electrons from the periplasmic electron carriers to extracellular electron acceptors (minerals or electrodes). (**B**) *Geobacter sulfurreducens*: both inner heme-containing membrane proteins (ImcH and CbcL) are proposed to oxidize quinols. Electrons are transferred to periplasmic c-type cytochromes. The PpcA and PpcD are the most abundant c-type cytochromes. The latter finally transfer electrons to OmaB-OmbB-OmcB or OmaC-OmbC-OmcC porin–cytochrome trans-membrane protein complexes and then to extracellular electron acceptors (minerals or electrodes) [10,58,62].

*Shewanella oneidensis* strain MR-1 and two species of *Geobacter* (*G. sulfurreducens* DL-1 and *G*. *sulfurreducens* PCA) were the first bacteria identified as being able to use minerals that contain Fe(III), Mn(III), or Mn(IV) as terminal electron acceptors [67]. Further studies on *Shewanella* revealed the direct involvement of six multiheme cytochromes and porin-type outer membrane proteins in EET. Cytochromes form a trans-outer membrane protein complex, which transfers electrons from the periplasmic proteins to the bacterial surface [68,69,70,71]. On the bacterial surface, the porin-type proteins can physically transfer electrons directly to minerals that contain Fe(III), as shown in Figure 1A.

Several studies have reported that outer membranes possess nanowires that can mediate the transfer of electrons to minerals and make possible physical connections with neighbouring cells [44,72,73,74].

In a similar way, the EET system in *Geobacter* (Figure 1B) involves multiheme type-c cytochrome and porin-like outer membrane proteins. However, the number of proteins potentially involved seems to be higher (Figure 1B). Interestingly, *Geobacter* has been found to exchange electrons with bacteria of the same or different species, minerals, and electrically conductive carbon materials (activated carbon, biochar, and carbon cloth) [58]. For example, Summers [75] found the *Geobacter sulfurreducens* PCA strain used nanowires and their associated multiheme c-type cytochrome for accepting electrons from *Geobacter metallireducens* GS-15.

The same *G*. *metallireducens* GS-15 strain has also been found to transfer electrons directly to methanogenic *Archaea* through nanowires [76].

The extracellular electron transfer mechanisms identified in *Geobacter* and *Shewanella* are similar; however, the fact that these bacteria are not phylogenetically related suggests that these functions evolved independently through convergent evolution.

## 4. Electrogenic Bacteria in Microbial Fuel Cells

The properties of the anode materials are one of the important aspects for the electricity production. Carbon-based materials, such as graphite fibre brushes, rods, felts, and fabrics, are used to design electrodes due to their high performance, low cost, strong biocompatibility, and high electrical conductivity [77]. Some authors recently mixed a soybean–potato powder with conductive materials to produce an anode able to promote biofilm growth on it [78]. Other natural materials (e.g., carbonized sponge-like natural pomelo peel or carbonized neem wood, subsequently modified) and metallic compounds have also been used and tested [79], although most of them have not been applied in full-scale MFCs so far.

Electric power (P) is generated by electron flows from anode to cathode. Power generation is an essential parameter for estimating the performance of a microbial fuel cell. P (measured in mW) is calculated in accordance with Ohm’s law. P is directly related to voltage (V, measured in mV) and it is indirectly dependent on electrical resistance (R, measured in ohms, Ω):

P = V^2^/R

Some authors report electrical performance of MFCs in terms of Power density (PD), considering the surface (A) of the anode/cathode (W/m^2^) as follows [80]:

PD = P/A

In most cases, electrical measurements are acquired with commonly available voltage meters, multimeters, and measurement acquisition systems connected in parallel with the MFC circuit. Measurement acquisition systems can modulate operating conditions by switching from open to closed-circuit conditions and vice versa, varying resistance and modulating the charge (open circuit) and discharge (closed circuit) period [21].

## 5. MFCs for Wastewater Contaminant Removal

Electrogenic bacteria can be exploited for contaminant removal and bioremediation processes by designing suitable microbial fuel cells. EAB releases electrons that can oxidize and transform organic matter and contaminants in water rich in organic carbon, wastewater, soil, or sediment [81]. Over the past few years, MFCs have shown a promising sustainable technology for simultaneous energy generation and wastewater treatment, with reduced solid waste production compared to conventional aerobic processes [51]. Indeed, aerobic wastewater treatment is an energy-demanding process producing large amounts of residual solids, which are costly to treat and dispose [82].

Some authors [83] have recently reviewed some mathematical models able to predict microbial growth and substrate biodegradation for optimizing and controlling bioprocesses and MFC design. The possibility of modelling the bacterial gene regulatory network could be a good approach for improving knowledge on electroactive bacteria, and it may be a starting point for designing more efficient MFCs and are essential for their scaling up.

Liu et al. [84] reported one of the first experiments with a single chamber MFC for treating domestic wastewater and obtained a total chemical oxygen demand (COD) removal of about 50%. Ye et al. [85] tested a dual-chamber MFC, including an anode chamber, using anaerobic sludge from a wastewater treatment plant. The system obtained about 90% of COD removal at 150 days of operation and a maximum voltage generation of 598.9 mV. Dairy wastewater in a dual chamber MFC was also used as the electrolyte, as shown by Sanjay et al. [86], and reached stable electric generation and a total COD reduction of 95.2%.

MFCs can decrease not only COD but also nitrate and heavy metal concentrations in industrial wastewaters. For example, MFCs have been used to co-treat wastewater from an industrial acid mine drainage (I-AMD), rich in heavy metals and with a low pH, with municipal wastewater (MWW) [87]. For this purpose, a dual chamber MFC was filled with 50-50 of I-AMD and MWW and removal rates of Cd (42%), Cu (84%), Fe (71%), Al (77%), Pb (55%) and As (42%) were obtained. Moreover, a reduction in total nitrate (higher than 90%) was achieved.

Moreover, Tacas et al. [88] tested an acclimated inoculum, obtained by mixing aliquots of bacterial communities from a pristine hot spring, soil, water and pond water, in a dual-chamber MFC. This MFC system was able to degrade more than 90% of a mixture of three azo dyes (Sunset Yellow, Allura Red, and Tartrazine) in a few hours. The mixed consortium was identified by amplification of the 16S rRNA by Illumina Miseq. A co-dominance of *Klebsiella* spp. and *Enterococcus faecalis* was found, in accordance with their capability to degrade azo dyes. The occurrence of *Klebsiella* was also associated with its known electrogenic activity [89,90].

It is generally known that emerging contaminants (e.g., antibiotics) are not removed from traditional wastewater treatment plants [91]. In this context, Zang et al. [92] tested the possible degradation of the nitroaromatic antibiotic chloramphenicol in dual-chamber MFCs, separated by cationic exchange membranes. They inoculated an activated anaerobic sludge microbial consortium at the anodes and added acetate as an electron donor.

A degradation of 84% of this antibiotic (50 mg/L as it initial concentration) was found at 12 h. The anode biofilm community was identified and the exoelectrogenic *Azonexus* and *Comamonas* bacteria were the dominant genera. Moreover, antibiotic degrading bacteria, such as *Azoarcus*, *Rhodococcus*, *Comamonas*, *Nitrososphaera*, and *Chryseobacterium*, were identified.

The degradation of sulfamethoxazole and its metabolite 3-amino-5-methylisoxazole (20 mg/L) was demonstrated by Wang et al. [93]. The antibiotic degraded 85% within 12 h in a dual-chamber MFC separated by a cation-exchange membrane. In this case, an inoculum of sludge collected from a thermostatic anaerobic digester was used. In this work, the microbial community was not identified. More recently, Cheng et al. [94] studied antibiotic degradation in single chamber MFCs maintained for 2 months. They used an inoculum of bacteria from a sewage treatment plant, supplied with sodium acetate and phosphate buffer solution with different antibiotic concentrations of up to 40 mg/L of sulfamethoxazole. The highest power density (821 mW/m^2^) and voltage (709 mV) were obtained using 20 mg/L of the antibiotic. However, the highest removal rate (96%) was found with the highest antibiotic concentration (40 mg/L). In the same work, they also identified the laccase enzyme as involved in the antibiotic degradation. *Geobacter* was dominant in the MFCs with the lowest antibiotic concentrations, presumably because it resisted the antibiotic effect.

Other authors [95] tested two-chamber MFCs for degradation of the antibiotic sulfamonomethoxine. They found 92% removal of 10 mg/L of this antibiotic. Adding the antibiotic, the microbial community changed at the anode. In fact, a shift from electroactive bacteria (e.g., *Spirochaetaceae*, *Petrimonas*, *Acidovorax*, and *Geobacter*) to bacteria with dual functions of electricity generation and antibiotic degradation (*Cupriavidus*, *Rhodococcus*, *Sphaerochaeta*, and *Cloacibacillus*) was observed.

There are few works that report study cases of microbial fuel cells at full scale, which treat a high amount of wastewater. MFC scale-up and commercialization poses problems such as high construction costs, difficulty in developing high power structures, the MFC lifetime, and maintenance of a high level of efficiency, with difficulties in balancing yields with overall system upscaling [96,97]. A full scale MFC system was tested by Liang et al. [98] and operated for 1 year. It was a 1000 L modularized MFC system for treating municipal wastewater. This system comprised 50 stacked MFC modules, each with a volume of 20 L and equal dimension made of polyvinyl chloride (PVC) and separated by cation exchange membranes. Both electrodes were of granular activated carbon to maintain a large ratio of electrode surface area to the reactor volume. The MFC system provided removal rates of COD from 70% to 90%, power outputs in the range of 0.42–3.64 W/m^2^, and an energy recovery of 0.033 kWh per m^3^ of municipal wastewater. This work did not report any information about the EAB involved in it.

Blatter et al. [99] set up a stretched 1000-L MFC for treating municipal wastewater. The system (64 MFC modules) was made with transparent polyester and the electrodes were of reticulated vitreous carbon. COD was removed from 80% to 95%, generating from 0.015 to 0.060 kWh/m^3^ of wastewater. It was the highest value for a scaled-up MFC fed with municipal wastewater. The microbial community of the anode biofilm was studied by sequencing 16S rRNA with Miseq Illumina. The electrogenic *Geobacter* was dominant (>60%) at the anode and other microorganisms (e.g., nitrifying and denitrifying bacteria) increased when *Geobacter* lost its dominance due to the substantial COD removal.

## 6. MFCs for Bioremediation of Persistent Organic Pollutants (POPs) and Heavy Metals

MFCs can also be applied for remediating solid matrices (soil and sediment) from organic and inorganic contaminants. In this case, soil or sediment are the electrolyte, and they are termed terrestrial microbial fuel cells (TMFCs). TMFCs are more complex if compared to other MFCs where the electrolyte is a liquid (e.g., water or wastewater). Owing to soil and sediment heterogeneity and their variable abiotic factors (e.g., pH, texture, organic carbon, and water content), TMFC performance can vary significantly [15,100]. In particular, organic matter content can be a limiting factor in MFC electricity production and durability as well as in decontamination process effectiveness [101]. The efficiency of TMFC is generally lower than conventional liquid-based ones. Indeed, a conventional MFC fed with wastewater can produce up to 1300 mW/m^2^ [102,103], unlike TMFCs, which show lower performances.

Adding an external carbon source, such as glucose or compost, performance of terrestrial MFC electroactive bacteria can significantly improve [21,104,105,106]. Among abiotic factors, water content is another key limiting parameter for an appropriate functioning of this technology [107]. For example, if soil moisture is not suitable for electron transfer due to water evaporation, the power output decreases. For this reason, soil water content needs to be maintained constant to ensure anaerobic conditions at the anode and solute migration. In fact, when TMFCs are used for restoring contaminated soils or sediments, moisture needs to be close to its maximum water holding capacity [108]. For example, degradation of polycyclic aromatic hydrocarbons (PAH) [109,110] and other persistent organic compounds (POPs) [108] has been obtained in waterlogged soil. A recent work reports a petroleum refinery wastewater treatment with dual chamber MFCs, adding fermented milk as a co-substrate for improving hydrocarbon and COD removal. The substrate degradation rate was reported as COD/m^3^-day and its maximum value (0.420 kg COD/m^3^-day) was found for 80 petroleum refinery wastewater/20 fermented milk. In this work, the microbial communities operating in these MFCs have not been analysed [111].

In another study, Cao et al. [112] tested the degradation of hexachlorobenzene (HCB) in spiked (40–200 mg/kg) soil microbial fuel cells. The TMFCs were inoculated with an anaerobic sludge, and with more than 51% of soil moisture, they obtained the highest removal (71.15%); with lower soil water content, HCB removal decreased significantly (38.92%), showing that this parameter is a key factor. HCB degraded via the reductive dechlorination pathway under anaerobic conditions. The presence of the electrogenic bacteria *Geobacter sulfurreducens* and *Betaproteobacteria* was detected with the fluorescence in situ hybridization (FISH) technique. *G. sulfurreducens* was found between 10^5^–10^7^ cells/g soil, depending on HCB concentration.

Hao et al. [110] recently reviewed degradation of PAH in soil and sediment MFCs. Although they report several MFC laboratory studies, numerous gaps in knowledge of PAH degradation are still present. For example, the distinction between electrochemical bacteria and PAH degraders is not clear in most works. Moreover, the amount of PAHs adsorbed by soil/sediment, which can hamper their biodegradation, has never been discussed. A role of other organisms (e.g., algae, protozoa) in increasing microbial degradation of PAHs is not excluded. Ultimately, syntrophy, competitive or parasitic behaviour, which can be promoted by electron exchange between microorganisms in the complex biofilm microbial communities, has to be investigated.

Borello et al. [21] tested the degradation of the organochlorine pesticide 2,2-bis (p-chlorophenyl) 1,1-dichloroetylene (DDE) using microbial fuel cells with soil amended with compost to improve the organic carbon source. The overall results showed that TMFCs promoted a substantial (ca. 40%) DDE removal in 2 months compared to un-amended control cells, with peaks of voltage generation at 540 mV with compost present. Compost stimulated microbial activity and cell performance. The maximum power output was 55 mW and decreased strongly over time, in line with a decrease in microbial abundance and microbial activity, presumably due to a decrease in organic carbon and soil water content.

In another work, Li et al. [113] set up microbial fuel cells using marine sediment polluted by petroleum hydrocarbon (PHC). These MFCs efficiently degraded PHCs with an average from 39.7% to 48.3%. Wang et al. [114] tested MFCs for heavy metal decontamination using an agricultural soil contaminated artificially with copper nitrate (Cu(NO_3_)_2_). They demonstrated a migration of copper from the anode to the cathode thanks to the electrical process occurring in the cell. The electrical field between the electrodes made possible Cu^2+^ migration and precipitation as copper oxide (Cu_2_O) at the cathode, obtaining at the same time a maximal voltage and power density of 539 mV and 65.77 mW/m^2^, respectively.

## 7. Plant-Microbial Fuel Cells and Constructed Wetland MFCs

Another type of microbial fuel cells is plant microbial fuel cells (Plant-MFCs). They are designed to combine MFCs with plants with different photosynthesis processes (C3, C4 and CAM) [115,116,117,118]. Plant-MFCs are a particular configuration of MFCs, where the electrolyte is water or sediment/soil (in saturated conditions) and plants are added for increasing electrical output and contaminant removal and/or organic matter degradation. Tubular or flat-plane models are the most common configurations [119]. The tubular model Plant-MFCs are the most studied and consist of a tube-shaped anode bordered by a membrane and then a cathode; several connections are placed into soil/sediment. Materials such as glass tubes/beakers, plastic containers, and polyvinyl chloride (PVC) are commonly used to fabricate Plant-MFCs. These systems provide both oxygen to the final electron acceptor (cathode), and organic substances in form of rhizodeposits, root exudates, and root border cells [120]. Exudates, key components of the rhizosphere, positively affect root colonization by microorganisms and enhance the metabolic activity of EAB [120]. Plants provide significant amounts of carbon, as rhizodeposits, and up to 60% has been estimated to be used as an energy source for microorganisms. A key factor is root development in the anode compartment in submerged and anaerobic conditions [121,122].

Plant-MFCs have been used for reducing the organic load of wastewater effluents because they are able to produce electricity more constantly and remove higher organic matter loads [96,120]. A current of 26 ± 7 mW/m^2^ anodic geometric area was obtained in a sediment microbial fuel cell, with rice plant presence, seven times higher than in their absence; power production of up to 330 W/ha was calculated for the oxidation of the rhizodeposits [123].

Plant-MFCs have also been used for contaminant removal (e.g., hydrocarbons and heavy metals) from soil or sediments. For example, using a soil spiked with Cd(II) (20 mg/kg), *Oryza rufipogon*, and *Typha orientalis* were used [124]. Plant uptake of Cd in roots had a significant role in its remediation. A Cd remediation of 22–30% from soil, with maximum voltages of 137.12–350.50 mV, respectively, was obtained. Moreover, when adding supplementary organic carbon (chestnut biochar), Cd removal increased. *Anaeromyxobacter*, *Geobacter*, *Phenylobacterium*, and *Azospirillum* (Proteobacteria) were the dominant genera in the anode region.

The degradation (75–87%) of high molecular weight PAHs, such as pyrene (3.2 mg/kg) and benzo[a]pyrene (1.7 mg/kg), was proven in a contaminated sediment using a Plant-MFC with the *Acorus calamus* wetland species. The microbial community characterization of the anode biofilms highlighted the dominance of the *Geobacter*, *Desulfuromonas*, *Longilinea*, and *Bellilinea* genera. The facultative denitrifying bacterium *Denitratisoma* (which can use oxygen if available) both protected *Geobacter* (an oxygen sensitive bacteria) from O_2_ presence at the anode and was simultaneously able to degrade organic contaminants.

In a Plant-MFC with sediment spiked with pyrene and phenantrene and planted with the submerged macrophyte *Vallisneria spiralis*, a removal of these contaminants of 88% at 65 days was observed [125]. *Bacillus* and *Clostridium* (*Firmicutes*) and *Geobacter* were the genera most enriched in the anode biofilm, under closed circuit conditions.

Table 2 reports various plant species used for Plant-MFCs. Overall, promising results have been obtained with this type of MFC; however, only a few studies have been performed on real contaminated soil or sediment. Although it is expected that exoelectrogenic and degrading bacteria work in cooperation, their specific functioning and relationships have not been thoroughly investigated so far.

Constructed wetlands (CWs) are engineered systems designed and constructed to exploit natural processes involving common wetland vegetation, such as *Typha latifolia* (bulrush), *Elodea nuttallii* (western waterweed), *Cyperus papyrus* (Nile grass), *Canna indica* (purple arrowroot), *Phragmites australis* (common reed), soils or sediments, and their associated microbial communities to treat wastewater and/or improve water quality [132]. Constructed wetland is a broad ecotechnology including various technical solutions [133] and designed to simulate natural wetlands (nature-mimicking), but operating within a more controlled environment [134]. CWs have been used to treat wastewater from various origins, such as industrial, municipal, agricultural, storm wastewater, and run-offs [135,136]. However, CW treatment efficiency can be moderate for organic contaminants and inefficient for nutrient removal because of the unavailability of suitable terminal electron acceptors such as oxygen in a major fraction system in the bottom area [137]. CWs are structurally similar to single chamber MFCs: there is an upper aerobic zone and an anaerobic zone at the bottom of the system. This structural similarity makes them compatible for integration [137]. In fact, in the last decade, considerable progress has been made in integrating MFCs into constructed wetlands in the so-called CW-MFC system. In this case, the anode (electron acceptor) is buried in the deep anaerobic region of the CW, while the cathode is located in the surficial aerobic zone of the same CW. Both anode and cathode are connected by an external circuit. This new technology, taking advantage of electrogenic bacteria on a large scale, can be a cost-effective method for producing energy during the biodegradation of organic matter [138].

The first application of a CW-MFC was described by Yadav in 2012 [139], demonstrating the feasibility of simultaneous bioelectricity generation and treatment of textile industrial wastewater containing different concentrations of methylene blue dye in a small scale CW-MFC set up (internal diameter of 10.5 cm and length of 62 cm). A maximum of 93% dye removal was achieved at 96 h of treatment and a reduction of 75% of COD from wastewater was observed. The maximum power density and current density measured by an external circuit was 15.73 mW/m^2^ and 69.75 mA/m^2^, respectively. In this work, no information about the microbial community involved in the microbial fuel cells was reported.

Lu et al. [140] characterized a microbial community by pyrosequencing a sediment sample from a CW-MFC with *Canna indica* as the vegetal species. They found a dominance of Proteobacteria (38%), followed by *Acidobacteria* (20%), *Actinobacteria* (9%), *Chloroflexi* (8%), and *Bacteroidetes* (7%). The *Geobacter* genus was found at 7.4% in the anaerobic zone of the CW-MFC, in accordance with its ecological functioning as an electroactive bacterium.

In another work, Xu et al. [141] detected several bacterial groups related to the nitrogen cycle in a small scale CW-MFC with *Phragmites australis* as the vegetal species. In particular, they identified the ammonia oxidizing group *Nitrosomonadaceae*, the nitrite-oxidizing bacterium *Nitrospira*, and other denitrifying bacteria, such as *Bacillus* and *Thauera.* They also detected the EAB *Geobacter* and *Desulfovibrio* bacteria in the anaerobic zone. They demonstrated CW-MFC to be a technology able to decrease both nitrogen content and chemical oxygen demand, with an average removal rate of 82%, producing 266 mV of voltage and with the highest power density of 3714 mW/m^2^. Although there are several studies on the use of CW-MFCs for wastewater treatment [142,143,144,145,146], few have reported organic contaminant degradation [147] and specific applications in full-scale systems. Further research and development is needed to explore the feasibility and practicality of such a system at a full-scale level. Furthermore, few works have investigated the microbial community structure occurring in CW-MFCs, and identified the role of specific electrogenic bacteria involved in the electricity production.

Both Plant-MFCs and CW-MFCs rely on synergistic actions, only partially unveiled, between microorganisms and plants, which can provide key ecosystem services such as soil and water decontamination [148]. Consequently, they can be used for contaminant bioremediation as nature-based solutions.

## 8. Conclusions

Natural EAB are present in various environments (soil, water, sediments, human gut, etc.), belong to several and, in many cases, phylogenetically distant groups, and can have different ecological roles. Most works on MFCs report results in terms of electrical performance (e.g., electric power and voltage) with an engineering approach, neglecting investigations of EAB functioning. Indeed, an ecological approach aimed at functional and phylogenetic identification of EAB and of specific biotic (intra and interspecific interactions) and abiotic conditions that can improve their activity in MFCs is desirable. Consequently, further studies on the ecology of these microorganisms are necessary. The effectiveness of this green technology for bioremediation purposes can be improved not only by using the most suitable materials and configurations but also by applying biostimulation (e.g., adding organic matter, plant species) or bioaugmentation strategies (e.g., inoculum of selected bacteria) to maximize EAB activity.

## Figures and Tables

**Table 1:** Electroactive prokaryotic cells (*Bacteria* and *Archaea*) found in natural and anthropic environments.

Species	Habitat	References
*Acidiphilium cryptum*	Coal/copper strip mine soil/sediment	[38,39]
*Acidithiobacillus ferrooxidans*	Acid mine drainage water	[40]
*Acinetobacter johsonii*	Marine water	[41]
*Alcaligenes faecalis*	Ubiquitous/wastewater	[42]
*Arcobacter butzleri*	Freshwater/seawater	[33]
*Brevibacillus agri*	Paper wastewater treatment sludge	[43]
*Clostridium ljungdahlii*	Anaerobic digesters	[44]
*Clostridium* spp.	Ubiquitous in environment	[44,45]
*Desulfuromonas* sp.	Salt marsh sediment	[30]
*Dietzia* sp.	River estuary sediment	[46]
*Enterococcus faecalis*	Human gut	[37]
*Escherichia coli*	Ubiquitous/wastewater	[47]
*Geobacter metallireducens*	Soil/sediment	[47]
*Geobacter sulfurreducens*	Soil/sediment	[8]
*Leptothrix* sp.	Aquatic environments/wastewater	[48]
*Methanobacterium palustre*	Hot springs/anaerobic digesters	[49]
*Methanococcus maripaludis*	Salt marsh sediment	[44]
*Nocardiopsis* sp.	Saline and alkaline soil/marine ecosystem	[50]
*Ochrobactrum anthropi* YZ-1	Wastewater	[51]
*Pyrococcus furiosus*	Salt marsh sediment	[52]
*Shewanella oneidensis*	Deep sea anaerobic habitats/soil	[53]
*Sporomusa ovata*	Sugar beet leaf (endophyte)	[53]
*Thioalobacter*	Salt marsh sediment	[30]
*Thiomicrorhabdus* spp.	Deep-sea hydrothermal vents	[25]
*Pleomorphomonas* sp.	Plant roots (endophyte)	[36,54]
*Rahnella* sp.	Plant roots (endophyte)	[36,55]
*Shinella* sp.	Sugar cane steam (endophyte)	[36,56]
*Staphylococcus aureus*	Human gut	[37]
*Streptococcus agalactiae*	Human gut	[37]
*Winogradskyella poriferorum*	Marine water	[41]

**Table 2:** Plant species commonly used for Plant-MFCs and their applications for heavy metals and pyrene removal.

Plant Species	Habitat, Experimental Time	Max. Voltage (mV)	Contaminant Removal (%)	Reference
*Typha orientalis* ** 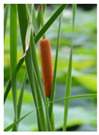 **	Soil, 150 days	137.12 ± 13.08	Spiked Cd(II), 20 mg/kg: 30.2%, mainly bioaccumulation in roots	[124]
*Oryza rufipogon* ** 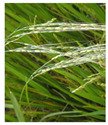 **	Soil, 150 days	350.50 ± 74.89	Spiked Cd(II), 20 mg/kg: 22.8%, mainly bioaccumulation in roots	[124]
*Oryza rufipogon* + Chestnut biochar (5%)		350.50 ± 74.89	Cd(II), 20 mg/kg: 31.7%	
*Pennisetum alopecuroides* *** 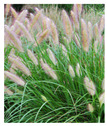 ***	Soil, 96 days	220 at day 0	Spiked Cr(VI), 50–500 mg/Kg: 75%	[126]
	Soil, 10 months		Spiked Cr(VI), 50 mg/kg: 65%	[127]
*Phragmites communis* *** 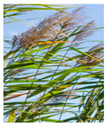 ***	Soil, 96 days	220 at day 0	Spiked Cr(VI), 50–500 mg/Kg: 75%	[126]
	Soil, 10 months		Spiked Cr(VI), 50 mg/kg: 78%	[127]
*Acorus Calamus* *** 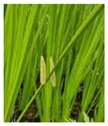 ***	Sediment, 367 days	61.4 mV (medium)	Pyrene, 3.2 mg/kg (87%) and benzo[a]pyrene, 1.7 mg/kg: 75%	[128]
	Sediment (Constructed wetlands)	36.43 mW/m^2^	Cr(VI), 12.07 mg/L in wastewater: 99%	[129]
*Vallisneria spiralis* *** 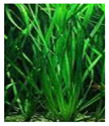 ***	Sediment	121.7 mW	Spiked Pyrene and phenantrene, 10 mg/kg: 88.2%	[125]
*Ipomoea aquatica* *** 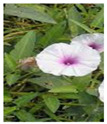 ***	Sediment	114 ± 5.89 mV	Spiked Cu, 170 mg/kg: 60%	[130]
*Lolium perenne* *** 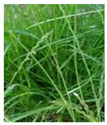 ***	Plant floated in water; 120–200 h	55 mA/m^2^	Cr(VI), 10–20 mg/L: 90%	[131]

## Data Availability

No new data were created or analyzed in this study. Data sharing is not applicable to this article.
